# Combined mitral valve replacement and coronary artery bypass grafting through a left thoracotomy after retrosternal oesophageal reconstruction

**DOI:** 10.1093/icvts/ivac161

**Published:** 2022-06-14

**Authors:** Ryosuke Numaguchi, Chikara Shiiku

**Affiliations:** Department of Cardiovascular Surgery, National Obihiro Hospital, Obihiro-city, Hokkaido, Japan; Department of Cardiovascular Surgery, National Obihiro Hospital, Obihiro-city, Hokkaido, Japan

**Keywords:** Mitral valve replacement, Coronary artery bypass grafting, Left thoracotomy

## Abstract

A 74-year-old man with a history of retrosternal oesophageal reconstruction was referred for surgical treatment of mitral valve regurgitation and coronary artery disease. He underwent mitral valve replacement combined with coronary artery bypass grafting through a left thoracotomy. Combined mitral valve replacement and coronary artery bypass grafting through a left thoracotomy were feasible in this patient with a retrosternal neo-oesophageal conduit.

## INTRODUCTION

Cardiac surgery in patients with a history of retrosternal oesophageal reconstruction remains challenging. We report a successful mitral valve replacement (MVR) combined with coronary artery bypass grafting (CABG) through a left thoracotomy in a patient with a retrosternal neo-oesophageal conduit.

## CASE REPORT

**Figure 1:**
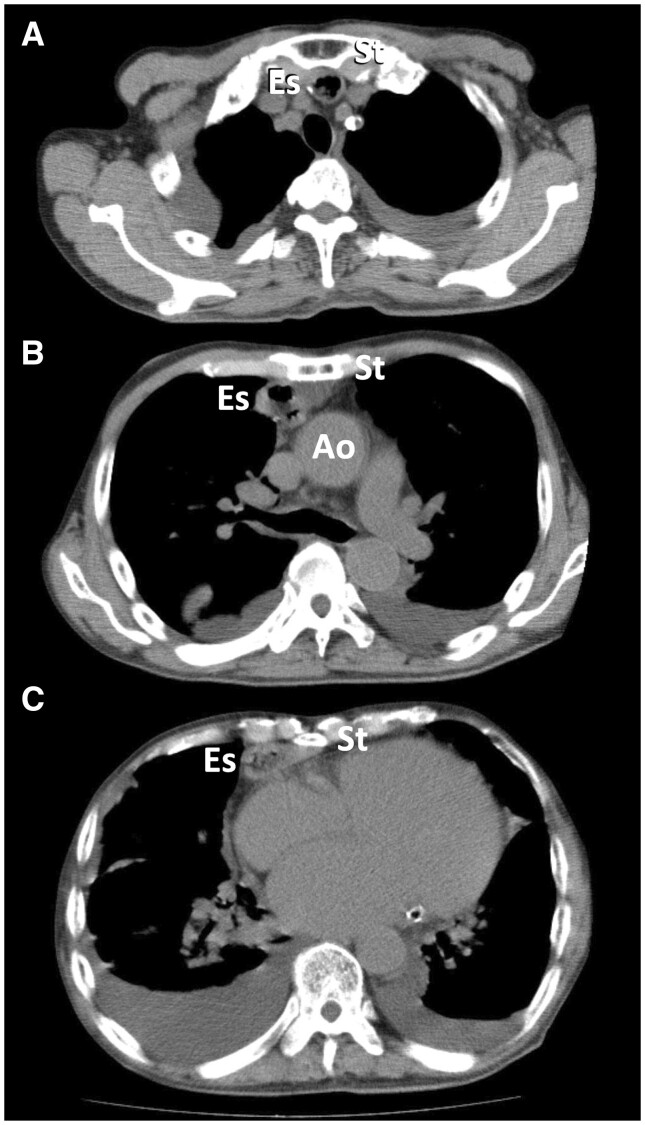
Computed tomography showing relationship among the reconstructed oesophageal conduit (Es), sternum (St) and ascending aorta (Ao) at the level of the (**a**) upper, (**b**) middle and (**c**) lower parts of the sternum.

**Figure 2:**
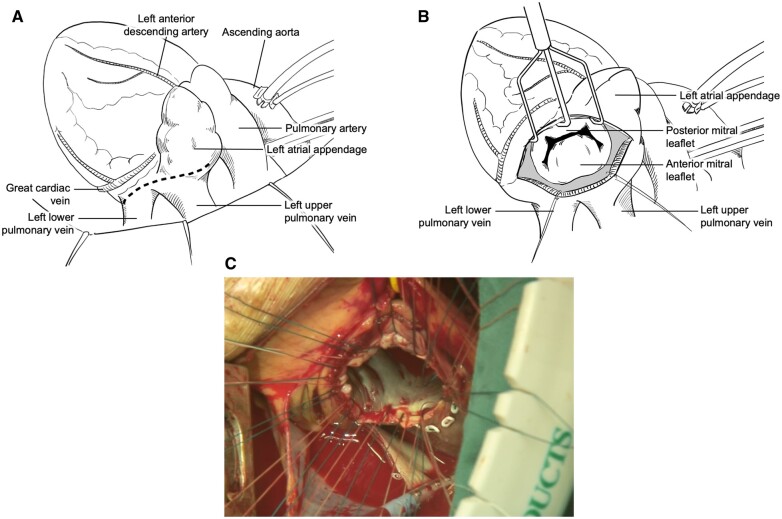
(**a**) Left atrial incision is shown by the dotted line. (**b**) The exposure of the mitral valve is upside down in contrast to that in the median sternotomy. (**c**) Surgical view of the mitral valve through the left thoracotomy after mattress suturing.

A 74-year-old man was referred for surgical treatment of mitral valve regurgitation and coronary artery disease. He underwent oesophageal surgery for oesophageal carcinoma with retrosternal gastric tube reconstruction through a right thoracotomy 11 years ago. Transthoracic echocardiography showed severe mitral valve regurgitation due to a flail mitral valve posterior leaflet and mildly reduced left ventricular systolic function with an ejection fraction of 43%. Coronary angiography revealed occlusion of the left anterior descending artery. Computed tomography revealed that the neo-oesophageal conduit was located just beneath the upper and lower parts of the sternum (Fig. 1) and that the bilateral iliac arteries were severely stenosed due to circumferential calcification. The median sternotomy approach was considered to be associated with a high risk of oesophageal injury. In addition, performing CABG through a right thoracotomy seemed difficult. Therefore, we decided to perform MVR and CABG through a left thoracotomy. The operation was performed with the patient under general anaesthesia with selective bronchial intubation. The patient was placed in the 45° semi-oblique right lateral decubitus position. The pleural cavity was opened at the fifth intercostal space by making a 15-cm skin incision. The left internal thoracic artery was harvested. Then the pericardium was opened longitudinally, anterior to the phrenic nerve. Cardiopulmonary bypass (CPB) was established with the ascending aorta inflow and the right femoral vein outflow. The ascending aorta was cross-clamped and cardioplegia was administered in an antegrade fashion. The left atrium was opened by making a longitudinal incision that started at the base of the left atrial appendage and extended to the left inferior pulmonary vein (Fig. 2a). The orientation of the mitral valve was inverted compared with that achieved by a median sternotomy or a right thoracotomy (Fig. 2b). The mitral valve was removed and replaced with a 31-mm St. Jude Medical Epic heart valve (St. Jude Medical, Inc., St. Paul, MN, USA) with excellent exposure (Fig. 2c). After closing the left atrial incision, CABG was performed during cardiac arrest. Air was carefully removed from the left ventricle; then the aorta was declamped. CPB and aortic cross-clamp times were 157 min and 109 min, respectively. Postoperative transthoracic echocardiography showed no paravalvular leakage, and coronary computed tomography angiography revealed that the bypass graft was patent. The postoperative course was uneventful. At the 6-month follow-up, the patient was doing well without any signs of heart failure.

## DISCUSSION

Cardiac surgery in patients with retrosternal neo-oesophageal conduits poses significant technical challenges associated with adequate access to cardiac structures and CPB strategies. Inra *et al.* reported 8 cardiac surgical cases after extra-anatomic oesophageal reconstruction (6, retrosternal; 2, subcutaneous) (1). They performed open cardiac surgery through a median sternotomy in 4 cases, a right thoracotomy in 2 and a left thoracotomy in 2 cases. They reported that 1 delayed injury to the retrosternal conduit occurred after the median sternotomy approach. This finding implies that a median sternotomy involves a potential risk of oesophageal conduit injury in patients with a history of retrosternal oesophageal reconstruction.

Mitral valve surgery through a left thoracotomy is rarely performed in selected patients. Suzuki *et al.* reported a case series of 16 patients who underwent the left thoracotomy approach for multiple redo mitral valve operations (2). All procedures were performed through the posterolateral fifth intercostal space. They reported that exposure of the mitral valve was uniformly excellent.

In this case, mitral valve repair was not performed mainly because the intraoperative transoesophageal echocardiography could not be used. In addition, when complex mitral valve repair was needed, the time required for CPB and aortic cross-clamping might have been extended because of our inexperience with this approach. Ultimately, we could perform MVR and CABG through a left thoracotomy without any difficulty. Combined MVR and CABG through a left thoracotomy was feasible in a patient with a retrosternal neo-oesophageal conduit.
